# Effects of diet and ovariectomy on *Toxoplasma gondii* brain infection: functional alterations and neuronal loss in rats

**DOI:** 10.1093/braincomms/fcae441

**Published:** 2024-12-31

**Authors:** Nene Ahidjo, Paul F Seke Etet, Leonard Ngarka, Frederic Maidawa Yaya, Ethel W Ndianteng, Aude L Eyenga Nna, Luc Yvan Meka’a Zang, Christelle Kemmo, Caroline N C Nwasike, Floriane G Yonkeu Tatchou, Wepnyu Y Njamnshi, Leonard N Nfor, Patrick V Tsouh Fokou, Sefirin Djiogue, Fabrice Fekam Boyom, Bonaventure T Ngadjui, Alfred K Njamnshi

**Affiliations:** Brain Research Africa Initiative (BRAIN), P.O. Box 25625, Yaoundé, Cameroon; Brain Research Africa Initiative (BRAIN), P.O. Box 25625, Yaoundé, Cameroon; Neuroscience Laboratory, Faculty of Medicine and Biomedical Sciences, The University of Yaoundé I, P.O. Box 25625, Yaoundé, Cameroon; Basic and Translational Research Unit, Center for Sustainable Health and Development, Garoua, Cameroon; Department of Physiological Sciences and Biochemistry, Faculty of Medicine and Biomedical Sciences, University of Garoua, P.O. Box 346 Garoua, Garoua, Cameroon; Brain Research Africa Initiative (BRAIN), P.O. Box 25625, Yaoundé, Cameroon; Neuroscience Laboratory, Faculty of Medicine and Biomedical Sciences, The University of Yaoundé I, P.O. Box 25625, Yaoundé, Cameroon; Neuroscience Laboratory, Faculty of Medicine and Biomedical Sciences, The University of Yaoundé I, P.O. Box 25625, Yaoundé, Cameroon; Basic and Translational Research Unit, Center for Sustainable Health and Development, Garoua, Cameroon; Department of Physiological Sciences and Biochemistry, Faculty of Medicine and Biomedical Sciences, University of Garoua, P.O. Box 346 Garoua, Garoua, Cameroon; Neuroscience Laboratory, Faculty of Medicine and Biomedical Sciences, The University of Yaoundé I, P.O. Box 25625, Yaoundé, Cameroon; Neuroscience Laboratory, Faculty of Medicine and Biomedical Sciences, The University of Yaoundé I, P.O. Box 25625, Yaoundé, Cameroon; Neuroscience Laboratory, Faculty of Medicine and Biomedical Sciences, The University of Yaoundé I, P.O. Box 25625, Yaoundé, Cameroon; Laboratory of Animal Physiology, Faculty of Science, University of Yaoundé I, P.O. Box 812, Yaoundé, Cameroon; Neuroscience Laboratory, Faculty of Medicine and Biomedical Sciences, The University of Yaoundé I, P.O. Box 25625, Yaoundé, Cameroon; Laboratory of Animal Physiology, Faculty of Science, University of Yaoundé I, P.O. Box 812, Yaoundé, Cameroon; Neuroscience Laboratory, Faculty of Medicine and Biomedical Sciences, The University of Yaoundé I, P.O. Box 25625, Yaoundé, Cameroon; Neuroscience Laboratory, Faculty of Medicine and Biomedical Sciences, The University of Yaoundé I, P.O. Box 25625, Yaoundé, Cameroon; Laboratory of Animal Physiology, Faculty of Science, University of Yaoundé I, P.O. Box 812, Yaoundé, Cameroon; Brain Research Africa Initiative (BRAIN), P.O. Box 25625, Yaoundé, Cameroon; Neuroscience Laboratory, Faculty of Medicine and Biomedical Sciences, The University of Yaoundé I, P.O. Box 25625, Yaoundé, Cameroon; Division of Health Operations Research, Ministry of Public Health, P. O. Box 1937, Yaoundé, Cameroon; Brain Research Africa Initiative (BRAIN), P.O. Box 25625, Yaoundé, Cameroon; Neuroscience Laboratory, Faculty of Medicine and Biomedical Sciences, The University of Yaoundé I, P.O. Box 25625, Yaoundé, Cameroon; Antimicrobial & Biocontrol Agents Unit, Laboratory for Phytobiochemistry and Medicinal Plants Studies (LPMPS), The University of Yaoundé I, P. O. Box 812, Yaoundé, Cameroon; Department of Biochemistry, Faculty of Science, University of Bamenda, P.O. Box 39, Bamenda, Cameroon; Advanced Research and Health Innovation Hub, P.O. Box 20133, Yaoundé, Cameroon; Laboratory of Animal Physiology, Faculty of Science, University of Yaoundé I, P.O. Box 812, Yaoundé, Cameroon; Antimicrobial & Biocontrol Agents Unit, Laboratory for Phytobiochemistry and Medicinal Plants Studies (LPMPS), The University of Yaoundé I, P. O. Box 812, Yaoundé, Cameroon; Advanced Research and Health Innovation Hub, P.O. Box 20133, Yaoundé, Cameroon; Department of Organic Chemistry, The University of Yaoundé I, P.O. Box 812, Yaoundé, Cameroon; Brain Research Africa Initiative (BRAIN), P.O. Box 25625, Yaoundé, Cameroon; Neuroscience Laboratory, Faculty of Medicine and Biomedical Sciences, The University of Yaoundé I, P.O. Box 25625, Yaoundé, Cameroon

**Keywords:** *Toxoplasma*, low-protein diet, high-fat diet, ovariectomy, behaviour

## Abstract

Epidemiological evidence associates *Toxoplasma gondii* latent infection with the development of neuropsychiatric disorders, and various immunological and environmental factors play key pathophysiological roles through host immune response alterations. We investigated the cognitive and motor alterations occurring in the terminal stage of *T. gondii* infection in rats, and whether a low-protein diet, a high-fat diet or ovariectomy may accelerate their development, given the role of malnutrition and menopause on immunity and resistance to infection. In two sets of experiments, 2-month-old (157.5 ± 4.3 g, *n* = 42) male (*n* = 18) and female (*n* = 24) Wistar rats were infected with *T. gondii* (ATCC 40050). Open-field and elevated plus maze tests were performed in the terminal stage of infection first and then in the early stage in low-protein diet–fed, high-fat diet–fed and ovariectomized infected rats. Late-stage (90 days) infected and early-stage (17 days) low-protein diet–fed groups showed significant decreases in body weight (42.42%↓, *P* = 0.016 and 57.14%↓, *P* < 0.001 versus non-infected, respectively), increases in body temperature (*P* = 0.001 and *P* < 0.001, respectively), decreases in blood glucose levels (*P* = 0.006 and *P* = 0.020, respectively), signs of cognitive and motor impairment and lower neuron counts. The alterations observed in high-fat diet–fed and ovariectomized infected animals were milder. Low-protein diet feeding to *T. gondii*-infected rats accelerated the occurrence of the infection terminal stage. Thus, a diet low in proteins could transform a slow early-stage *T. gondii* infection into an active neurotoxoplasmosis with neuropsychiatric manifestations and possible neurodegeneration in rats.

## Teaser

Ahidjo *et al*. report that exclusive feeding with a low-protein diet for 17 days caused cognitive and motor signs typically observed in the terminal stage in rats recently infected with *Toxoplasma gondii*. Thus, undernutrition may transform an asymptomatic *T. gondii* infection into an active neurotoxoplasmosis with neurological and psychiatric manifestations in rats.

## Graphical Abstract

**Graphical Abstract:**
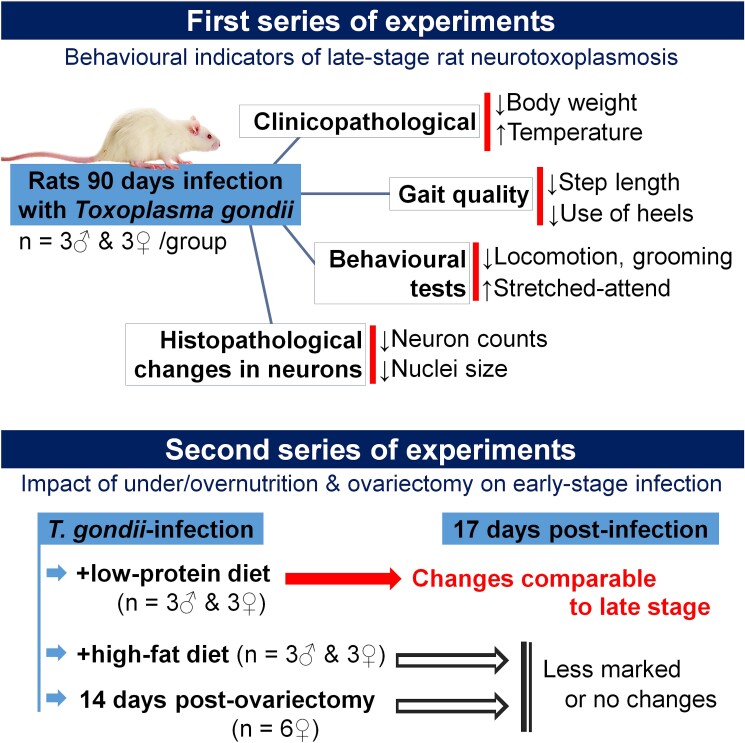

## Introduction

The apicomplexan parasite *Toxoplasma gondii* has infected more than a third of the world's population.^1,2^ In humans, while infections in developing foetuses and immunocompromised individuals result in a disease characterized by severe neuropsychological and ocular affections,^3-5^ until recently, infections of immunocompetent individuals were considered mild because they are usually asymptomatic.^6,7^ However, a growing number of human epidemiological studies,^8-10^ studies in experimentally infected laboratory rodents^11,12^ and *in vitro* studies^12,13^ suggest a potential causal relationship between *T. gondii* infection and the development of neurological, psychiatric and motor disorders in immunocompetent humans, including Alzheimer's disease, schizophrenia, bipolar disorder, movement disorders,^14-16^ cryptogenic epilepsy^17,18^ and even some brain cancers^19-21.^

Various immunological and environmental factors capable of affecting the immune response may have contributed to the global success of *T. gondii* infection, including undernutrition, overnutrition and menopause as described below. Undernutrition, particularly diets low in proteins that are common in low- and middle-income countries, negatively impact the metabolism and functions of immune cells.^22,23^ Early studies reported that infected rodents lost their resistance against *T. gondii* infection in case of zinc deficiency,^24^ vitamin D deficiency^25^ or even when starved.^23^ In addition, overnutrition is common in high-income countries and is associated with a dysregulation of the immune system.^26,27^ A positive association was reported between *T. gondii* seropositivity and obesity.^28,29^ Moreover, in menopause, ageing-associated immune function decline is worsened by oestrogen deprivation.^30,31^ Brain diseases associated with *T. gondii* infection would occur more often and be exacerbated during the peri-menopausal period in women, including migraine, anxiety and depression disorders, schizophrenia and cognitive decline.^32-35^

To evade immune responses, the *T. gondii* circulating form (tachyzoite) transforms into a drug-resistant and persistent encysted form (bradyzoite) that resides primarily in brain and muscle tissue^36,37^ and contributes to the development of cognitive and motor impairments in humans^6,38,39^ and in experimental models,^11,40,41^ at least partly through reactive neuroinflammation and resulting neurotransmitter imbalance.^14,15^*T. gondii* induces the downregulation of the central noradrenergic system in chronically infected rodents, with altered noradrenergic-associated behaviours of sociability and arousal,^13,42^ concomitantly with neuropsychiatric signs, including reduced sleep and increased wakefulness in adults^38,41^, and mood disorders, attention deficit, ocular and motor disorders and cryptogenic epilepsy in children.^43,44^

Furthermore, higher infection rates of *T. gondii* were reported in immunocompetent patients with unknown CNS diseases.^9,10^ In a recent study, anti-*T. gondii* IgG were observed in 25 patients with schizophrenia (55.6%) against 13 in controls (28.9%), and increased serum dopamine levels were observed among patients with schizophrenia.^39^ Thus, chronic *T. gondii* infection causes high dopamine levels that may contribute to the development of schizophrenia. Moreover, studies in *T. gondii*-infected mice revealed reduced glutamate and D-serine levels in prefrontal cortical and hippocampal tissue homogenates suggestive of glutamate and D-serine imbalance,^40^ as well as strong disruptions of gamma-aminobutyric acid and glutamatergic signalling pathways including schizophrenia-like downregulation of both metabotropic and ionotropic glutamate receptors.^45^ However, data supporting the actual ability of cystogenic strains of *T. gondii* to induce behavioural alterations and the timing and severity of such alterations are still needed,^7,40,46^ considering the mechanistic implications on the actual importance of cyst persistence for brain involvement.

We assessed the cognitive and motor responses of Wistar rats at the terminal stage of experimental infection with *T. gondii* TS-4 (ATCC 40050), a clinically relevant cystogenic mutant of the RH strain.^47,48^ We further assessed the ability of experimental undernutrition [low-protein diet (LPD)] and overnutrition [high-fat diet (HFD)] to accelerate the disease course in early-stage infected rats, to assess whether these factors contribute to the development of neurotoxoplasmosis and related neurological and psychiatric disorders. In addition, ovariectomized female rats, a well-established and widely used model of menopause,^49,50^ was used to measure the impact of menopause on the course of the infection.

## Materials and methods

### Animals

**Figure 1:**
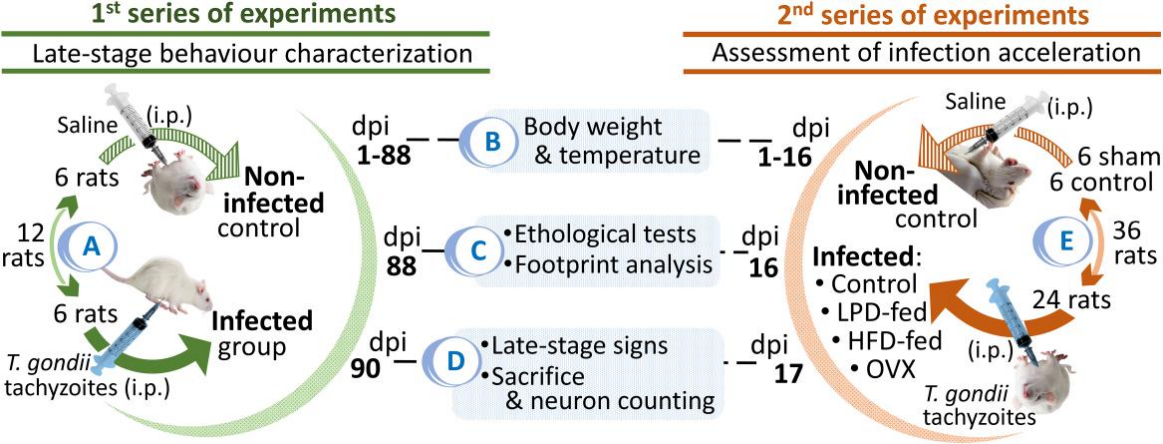
**Experimental procedures of the study.** In the first series of experiments, rats were infected (**A**), monitored until disease late stage and sacrificed (**B** and **C**). Brains were processed for neuron counting (**D**). In the second series of experiments, healthy or ovariectomized (OVX) rats were infected. Then, they were fed chow for OVX rats and either a LPD or a HFD for the others. Then, these rats were monitored and sacrificed when disease late-stage–like signs occurred in some animals (17 days) (**E**). For both experiments, behavioural tests were performed the day before sacrifice. i.p., intraperitoneal.

Two-months-old (157.5 ± 4.3 g, *n* = 42) male (*n* = 18) and female (*n* = 24) Wistar rats were obtained from the Faculty of Science of The University of Yaoundé I (Yaoundé, Cameroon) in two waves corresponding to the beginning of each of the two series of experiments performed (Fig. 1). They were acclimatized to the Neuroscience Laboratory conditions (Faculty of Medicine and Biomedical Sciences, The University of Yaoundé I). Animals had free access to water and food and were housed under a 12:12 light-dark cycle, at 25°C. All experimental procedures were approved by the institutional ethics committee (No. 0634/UY1/FMSB/VDRC/DAASR/CSD), and the animals were handled considering ethical rules relating to the protection of animals used for scientific purposes, particularly the European Commission Directive 2010/63/EU.

### Experimental procedures

**Figure 2:**
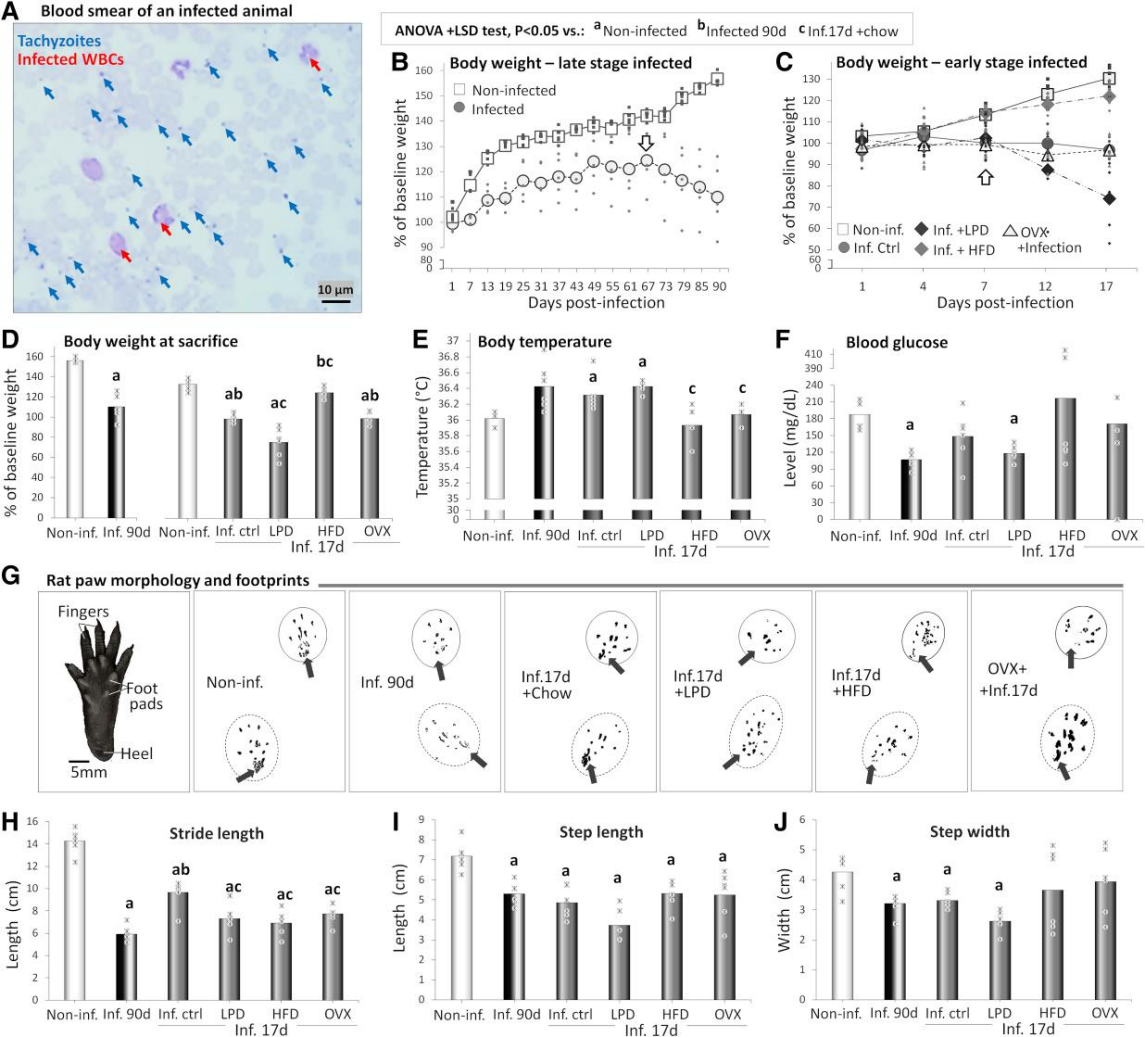
**Physiological parameters and gait quality indicators.** (**A**). Micrograph showing tachyzoites in Giemsa-stained blood smears. Body weights of non-infected (non-inf.) animals, *T. gondii*-infected animals at the terminal stage of infection (inf. 90d) (**B**) and infected animals at the early stage (inf. 17d) fed chow (inf. ctrl), LPD, HFD or ovariectomized (OVX) (**C**). Note curve inflections (arrows) on Day 67 post-infection for terminal-stage infected animals (**B**) and at Day 7 post-infection for infected rats fed LPD (**C**). Average body weight (**D**), body temperature (**E**) and blood glucose level (**F**) at sacrifice. Rat paw morphology and footprints of representative rats (**G**) and average stride lengths (**H**), step lengths (**I**) and step widths (**J**) of the same animals. Note that, unlike the non-infected, the infected animals use their anterior footpads more and their heels less. *n* = 6 animals/group. Critical value of ANOVA *F*-score: 4.35.

The first series of experiments was aimed at characterizing behavioural indicators of neurotoxoplasmosis occurring in the terminal stage of *T. gondii* infection in rats (Fig. 1). Hence, 12 animals were randomly divided in two groups (*n* = 6 per group, with 3 males and 3 females): (i) a non-infected control group injected with saline (i.p.) and (*ii*) a group infected with *T. gondii* tachyzoites (TS-4/ATCC 40050) kindly provided by the Antimicrobial & Biocontrol Agents Unit of The University of Yaoundé I. Rats were injected (i.p.) once with 10 million *T. gondii* tachyzoites in suspension in 500 µl of sterile saline, considering pilot studies in our laboratory and previous reports.^51,52^ The infection was confirmed when tachyzoites were observed in blood smears from the tail vein 3 days after inoculation, both inside and outside leucocytes^51^ (Fig. 2A). The animals were continuously monitored and video-recorded, and their body weight and body temperature (inner ear, infra-red thermometry) were measured every 3 days. Two ethological tests, the open-field test (OFT) and the elevated plus maze (EPM) paradigm, as well as footprint analysis were performed sequentially on day post-infection (dpi) 88, that is 20 days after the infected animals displayed an inflection in body weight (see the ‘Terminal-stage infected animals’ section). Thereafter, all animals were sacrificed under deep anaesthesia when signs of terminal disease were observed in infected animals (dpi 90), to avoid unnecessarily prolonging animal suffering. Brains were collected and processed for Nissl staining. Then, neuron nuclei were counted.

The aim of the second series of experiments was to assess whether undernutrition, overnutrition or ovariectomy could accelerate the early-stage of the infection into terminal-stage neurotoxoplasmosis (Fig. 1). Here, 30 animals were randomly divided into five groups (*n* = 6 females per group for the ovariectomized, and *n* = 3 females and 3 males pergroup for the other groups), of which a non-infected control group that was injected with saline (i.p.) and fed normal rat chow [15% protein, 3% fat and 7% simple sugars (w/w)], and four groups that were infected with *T. gondii* tachyzoites: (i) a group fed on normal rat chow (infected control group); (ii) a group fed on LPD [normal chow-like diet with 7% protein (w/w)]^53,54^; (iii) a group fed a HFD [15% protein, 16% fat and 33% simple sugars (w/w) prepared as described before]^55^ and (iv) a group ovariectomized and allowed 14 days to recover from surgery before infection as described before.^56^ An ovariectomized control group (non-infected) was also used in the experiments.

Then, as in the previous series, after confirming the success of the infection, the animals were continuously monitored and video-recorded, and their body weight and body temperature were measured every 3 days. Then, however, the OFT test, EPM paradigm and footprint analysis were performed sequentially 10 days after the occurrence of an inflection in the body weight curve of LPD-fed infected rats (see the ‘Animals sacrificed at early stage’ section). The day after (dpi 17), also to avoid unnecessary suffering, animals were sacrificed under deep anaesthesia. Brains were collected and processed for Nissl staining, followed by neuron counting.

### Ovariectomy

Female Wistar rats were bilaterally ovariectomized employing the dorsal approach and using standard procedures, as previously described.^56,57^ Briefly, animals were anaesthetized with valium (10 mg/kg, i.p.) and ketamine (50 mg/kg, i.p.). The anaesthesia was confirmed by a reduction in respiratory rate and the lack of response following gentle pinching of footpad. A ventral incision was made through the skin on the right flank, and the ovary, oviduct and top of the fallopian tubes were clamped and removed. The skin and abdominal walls were sutured, and animals were returned to their cages. Comparable surgical procedure was performed in sham-operated rats, but ovaries were just palpated, not removed. Animals were administered with carprofen (Rimadyl®, 5 mg/kg s.c.) and amoxicillin (Clamoxyl®, 150 mg /kg s.c.) for postoperative analgesia and antibiotic prophylactic therapy, respectively, and monitored daily until confirmation of post-surgical recovery. They were infected 14 days after surgery, when endogenous hormonal decline was effective, as shown in previous reports.^56,57^

### Open-field test

The open-field arena was a 50-cm high wooden box with 100 × 100 cm floor including a 40 × 40 cm central zone and a peripheral zone. In this test, a rat was placed facing the wall in a corner of the arena, and the animal's activity was video-recorded for 10 min. Vertical and horizontal activities were simultaneously video-recorded using a computerized camera (LifeCam Studio, Microsoft, Redmond, WA, USA) placed 130 cm above the arena with a 45° angle. The floor and walls of the arena were cleaned with 70% alcohol solution after each trial, to prevent bias due to olfactory cues. The distance travelled in the arena and the number of entries and time spent in the central zone and in corners were determined using motion tracking on image sequences in the Image Processing Toolbox® of MATLAB software (MathWorks, Natick, MA, USA). The characteristics of episodes of stretch-attend posture (SAP, hindpaws stationary while the body is stretched forward for more than 3 s), rearing (on hind paws and against the wall) and grooming were scored from video-recordings offline. Gait and head posture qualitative scores were also determined (0–9 based on the severity of alterations).

### EPM paradigm

The EPM apparatus was raised 50 cm above ground level and consisted of two open arms (50 × 10 cm, no wall), two arms (50 × 10 cm) enclosed by wooden walls (40 cm high) and a common central platform (10 × 10 cm). Each rat was placed on the central platform facing an open arm and behaviour was recorded for 5 min. The performance on the EPM was recorded using a computerized video recording system including a camera (LifeCam Studio) placed 150 cm above the centre of the apparatus. After each trial, the floor and walls of the EPM were cleaned with a 70% ethanol solution. Video recordings were scored offline for the number of entries, time spent and distance travelled in the arms, as well as episodes of head dipping above the open arm edge, grooming and rearing. An entry occurred when all four limbs were within an arm.

### Footprint analysis

The footprint analysis was performed for the assessment of motor abilities, in particular gait and balance.^58,59^ Rats with inked paws were allowed to walk freely along an enclosed box (50 cm long, 10 cm wide and 20 cm high walls) with a clean sheet of paper placed on the floor. For each animal, a valid trial over three consecutive trials was considered, in order to avoid abnormal patterns associated with the habituation phase.^60^ The footprint patterns and contacts were digitized with a high-resolution scanner (HP Scanjet Pro 3000s3, Hewlett Packard, Palo Alto, CA, USA) and analysed. The step width and length were determined using MATLAB Image Processing Toolbox® (MathWorks, Natick, MA, USA).

### Nissl staining and neuron counting

Brains were fixed for 8 h in Karnovsky's fixative (50% glutaraldehyde and formaldehyde in 0.2 M phosphate-buffered saline), paraffinized and cut entirely in the transversal plane (thickness 5 µm). The zones of interest were the brain anterior zone including the anterior cingulate cortex, the brain posterior zone including the posterior parietal cortex and the cerebellar zone including the dentate nucleus. Three sections for each zone were selected using systematic random sampling, i.e. starting from a random point, and then progressing with a fixed periodic interval (8 μm in this study). Then, the selected sections were processed for Nissl staining using standard procedures. Briefly, after deparaffinizing with xylene (×2 for 10 min), sections were hydrated by immersion in ethanol baths diluted with distilled water of decreasing concentration (100, 100, 96, 70 and 50%, 5 min for each step), stained by immersion in cresyl violet solution for 5 min (for 100 ml: 0.02 g of cresyl violet acetate and 0.25 ml of glacial acetic acid in distilled water), rinsed in three changes of distilled water, dehydrated by immersion in ethanol baths of increasing concentration (50, 70, 96, 100 and 100%), cleared in three changes of xylene and mounted with DPX® medium (reference 4458, Sigma-Aldrich, Burlington, MA, USA).

Then neuron nuclei were counted in: (i) the anterior cingulate cortex that is involved in action, emotion and memory, with a cardinal role in the control of the expression of contextual fear generalization^61,62^; (ii) the medial septal nucleus that is critical for learning and memory, which prevented sepsis-induced cognitive deficits in mice^63,64^; (iii) the posterior parietal cortex, which is an associative region comprising the primary somatosensory areas^65^; (iv) the peri-fornical zone of the lateral hypothalamic area, which is critical for various physiological functions, including the promotion and stabilization of active arousal and drive to eating^66,67^; (v) the cerebellar molecular layer, which contains inter-neurons that are key elements of cerebellar network computation and behaviour^68^; and (vi) the cerebellar dentate nucleus whose neuron loss accounts for cerebellar symptoms in various neurodegenerative disorders.^69,70^ Neuron nuclei counts were performed on micrographs (taken at ×40 objective) of the areas of interest using semi-automatic counting with ImageJ software. More specifically, neuron nuclei were counted automatically using Nucleus Counting macro of Image J software (NIH, Bethesda, MD, USA). Then, an observer manually checked the particles counted to ensure that they all were neuron nuclei and that nuclei crossing the lower and left borders were discarded. In addition to neuron nuclei counts, the average size of neuron nuclei was also determined using ImageJ software.

### Statistical analysis

Statistical significance of inter-group differences in body weight, body temperature and cognitive and motor indicators revealed by footprint analysis, the EPM and the OFT and differences in neuron counts and size were assessed using ANOVA followed by least square difference *post hoc* test for inter-couple comparisons (OriginPro 8 software, OriginLab Co, Northampton, MA, USA). Differences with *P* < 0.05 were considered statistically significant. Data are presented as mean ± single data point values.

## Results

### Clinicopathological observations and body weight

#### Animal observation

Unlike non-infected animals (including ovariectomized controls, which were comparable with their non-ovariectomized counterparts, hence not presented), changes in body weight occurred with increasing disease severity. In the first 3 weeks after the infection day, most infected animals did not display observable differences compared with their non-infected counterparts. Then, between dpi 22 and 70, infected animals increasingly showed cachexia and heightened startling responses, with vocalization at handling. Afterwards, these signs were intensified and more functional alterations of nervous system appeared, including impaired posture and gait, together with signs indicating severe systemic disease such as a shaggy and dirty fur, porphyrin deposit around the eyes and appetite loss. Then, due to an increase in the aforementioned disease signs to the ethically acceptable limit of animal suffering, two infected animals were sacrificed at 87 dpi and the remainder at 90 dpi.

As also observed in the first series of experiments with long-term infected animals, infected animals fed on normal chow (infected control animals) did not display signs of disease up to dpi 17 when they were sacrificed, despite a slight appetite loss. Similar observations were made in ovariectomized animals infected during this early stage of infection. Interestingly, all LPD-fed infected animals displayed marked cachexia from the end of the first week of infection to dpi 17 when they were sacrificed. By dpi 17, all animals in this group presented with shaggy fur, porphyrin deposit around the eyes and impaired posture and gait, with four animals also displaying heightened startling responses and vocalization at handling. In contrast, HFD-fed animals did not display any sign of disease and looked like non-infected control animals.

#### Terminal-stage infected animals

Figure 2B–D present the progression of the body weights (Fig. 2B and C) and the body weights at sacrifice (Fig. 2D) of *T. gondii*-infected animals at the terminal stage of infection and infected animals at the early stage fed with LPD, HFD or ovariectomized. The growth curve of non-infected rats sacrificed after 90 days of monitoring was positive and had three major epochs: a first one between Days 1 and 19 (curve equation: *y* = 9.56*x* + 94.25, *R*^2^ = 0.97), a second slower between dpi 25 and 61 (*y* = 1.34*x* + 130.61, *R*^2^ = 0.91) and a last one faster than the latter and slower than the first between dpi 67 and 90 (*y* = 4.04*x* + 136.52, *R*^2^ = 0.93; Fig. 2B). From dpi 7 onward, the body weight increase was statistically significant compared with the baseline week values (*P* = 0.008; Fig. 2B). Instead, while the growth curve of infected rats also showed three linear epochs parallel to non-infected animals, some differences were observed (Fig. 2B). Notably, the fastest increase was also observed between Days 1 and 19 (*y* = 3.11*x* + 95.23, *R*^2^ = 0.90), followed by an epoch of slower growth between dpi 25 and 61 (*y* = 1.17*x* + 114.63, *R*^2^ = 0.65; Fig. 2B). However, infected rats displayed lower increases in body weight than non-infected rats throughout the study, and an inflection point was observed around dpi 67, from which the body weight started to decrease linearly (*y* = −3.51*x* + 127.97, *R*^2^ = 0.99; Fig. 2B). Hence, the average body weight at sacrifice was significantly lower in infected rats than in the non-infected (*P* = 1.7E−04; Fig. 2D).

#### Animals sacrificed at early stage

On the other hand, for groups sacrificed at dpi 17, comparable linear increases in body weight were observed in the non-infected group (*y* = 7.0*9x* + 93.75, *R*^2^ = 0.97) and in the infected group fed with HFD (*y* = 6.1*7x* + 93.11, *R*^2^ = 0.96; Fig. 2C). From 7 dpi, the body weights of these groups were significantly higher than baseline values (*P* = 3.5E–05 and 1.6E–04, respectively; Fig. 2C). Linear decreases in body weight were observed from Day 4 for both the infected control group (*y* = −1.90*x* + 104.82, *R*^2^ = 0.91) and the infected ovariectomized rats (*y* = −1.68*x* + 101.85, *R*^2^ = 0.88; Fig. 2C). The body weights of these animals remained comparable with the baseline values, as indicated by the lack of a statistically significant difference (*P* > 0.05).

Instead, the body weight of the infected group fed with LPD showed a polynomial progression (*y* = −2.55 × 2 + 8.74*x* + 94.57, *R*^2^ = 0.92) with an inflection point at 7 dpi, from which a linear decrease 7-fold faster than that observed in the infected control started (*y* = −13.63*x* + 115.07, *R*^2^ = 0.98; Fig. 2C). Significant decreases in body weight were observed in LPD-fed infected animals compared with baseline values from dpi 4 (*P* = 0.011; Fig. 2C). The average body weight of the terminal-stage group was also lower than that of the non-infected group (42.42%↓, *P* = 0.016; Fig. 2D).

Except for animals fed with HFD, the average body weights of all the infected animals sacrificed at dpi 17 were significantly lower than that of non-infected animals (*P* < 0.0001), that is the infected control (35.09%↓), the animals fed with LPD (57.14%↓) and the ovariectomized rats infected (34.81%↓; Fig. 2D). Instead, the average body weight of animals fed with HFD was only slightly decreased compared with that of the non-infected group (10.65%↓ *P* = 0.294; Fig. 2D). Moreover, the average body weight was significantly higher in the non-infected group sacrificed after 90 days than in the non-infected group sacrificed after 17 days (20.78↑, *P* < 0.001; Fig. 2D).

### Body temperature and blood glucose level

Figure 2E and F present the average values of body temperatures (Fig. 2E) and blood glucose levels (Fig. 2F) of *T. gondii*-infected animals at the terminal stage of infection and infected animals at the early stage fed with LPD, HFD or ovariectomized. An increase in body temperature was observed in infected rats at the terminal stage compared with their non-infected counterparts, but the high inter-individual variability prevented statistical significance (*P* = 0.258; Fig. 2E). In addition, infected animals fed with HFD and ovariectomized animals infected displayed body temperatures comparable with those of non-infected animals sacrificed after 17 days (*P* = 0.456 and 0.447, respectively; Fig. 2F). Instead, infected control group and animals fed with LPD presented with significant increases in body temperature at sacrifice compared with the non-infected group (*P* = 0.001 and *P* < 0.0001, respectively) and with other infected groups sacrificed at dpi 17 (*P* < 0.05; Fig. 2E).

On the other hand, significant decreases were observed in infected rats at the terminal stage compared with the non-infected group sacrificed after 90 days (*P* = 0.006; Fig. 2F). Instead, blood glucose levels at sacrifice of all rats sacrificed at dpi 17 were comparable with non-infected group levels (Fig. 2F), except for animals fed with LPD that displayed significant decreases (*P* = 0.020 versus non-infected group). In addition, unlike for the average body weight (see the ‘Terminal-stage infected animals’ section), the values of other parameters assessed in this study, including the body temperature, were comparable between non-infected groups sacrificed after 17 and 90 days (data not shown).

### Gait quality indicators

Figure 2G–J shows gait quality indicators of *T. gondii*-infected animals at the terminal stage of infection and infected animals at the early stage fed with LPD, HFD or ovariectomized. As shown for representative cases, the analysis of footprints revealed that compared with the non-infected animals (Fig. 2G), *T. gondii-*infected animals used their anterior footpads more and their heels less during locomotion, particularly at the front limbs, an alteration particularly marked in terminal-stage–infected animals and in infected animals fed with LPD (Fig. 2G). Significant decreases were observed in the average lengths of strides (Fig. 2H) and steps (Fig. 2I) of all infected groups compared with the non-infected group (*P* < 0.001). However, stride length decreases in the other infected groups were more marked than the decrease in the infected control group (*P* < 0.01; Fig. 2H). Instead, the decrease in step length was comparable between all infected groups, except for animals fed with LPD that displayed statistically lower lengths compared with infected control group (*P* = 0.026), animals fed with HFD (*P* = 0.05) and ovariectomized animals (*P* = 0.046; Fig. 2I). Moreover, unlike in animals fed with HFD and ovariectomized animals where high inter-individual differences were observed, the widths of the steps were significantly lower for terminal-stage–infected animals, the infected control group and animals fed with LPD compared with the non-infected control group (*P* = 0.029, 0.002 and <0.001, respectively; Fig. 2J).

### OFT cognitive and motor indicators

#### Locomotion episodes

**Figure 3:**
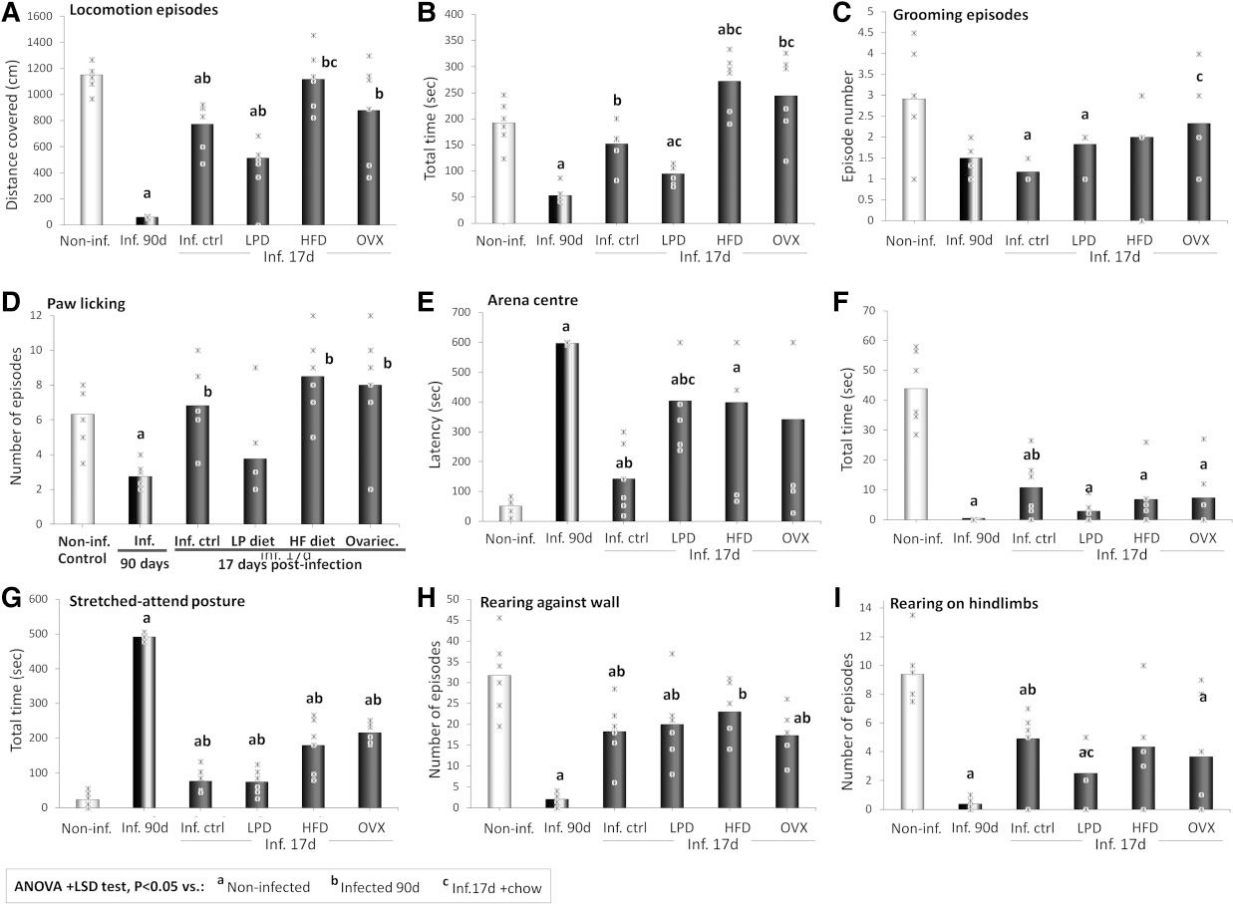
**OFT motor and cognitive indicators.** Distance covered (**A**) and locomotion time (**B**), grooming episode number (**C**), paw licking episode number (**D**), arena centre latency to first entry (**E**) and time (**F**), SAP time (**G**), rearing against the wall episode number (**H**) and rearing on hindlimbs’ episode number (**I**) in the open-field arena of non-infected (non-inf.) animals, *T. gondii*-infected animals at the terminal stage of infection (inf. 90d) and infected animals at the early stage (inf. 17d) fed chow (inf. ctrl), LPD, HFD or ovariectomized (OVX). *n* = 6 animals/group. Critical value of ANOVA *F*-score: 4.35.

Figure 3A and B present the distance covered (Fig. 3A) and the locomotion time (Fig. 3B) in the OFT arena of animals in the terminal stage of *T. gondii* infection, as well as the impact of ovariectomy, LPD and HFD on these parameters in animals in the early stage of infection. Compared with their non-infected counterparts, animals in the terminal stage of infection displayed significant decreases in the distance covered (*P* = 0.01; Fig. 3A) and in the total time spent walking (*P* < 0.001; Fig. 3B) in the arena.

The infected control rats sacrificed at dpi 17 and the infected rats fed with LPD also showed significant decreases in the distance covered compared with non-infected animals (*P* < 0.001), unlike for the groups of infected animals fed with HFD (*P* = 0.665) or ovariectomized (*P* = 0.133) where high inter-individual variabilities were observed (Fig. 3A). However, infected control rats had markedly higher numbers of episodes than the infected rats fed with LPD (*P* = 0.051), which in turn had a higher number of episodes than rats sacrificed in the terminal stage of infection (*P* = 0.005; Fig. 3A). Similar results were observed with the locomotion total time, although in addition the infected rats fed with HFD showed a statistically significant increase compared with the non-infected group (*P* = 0.026; Fig. 3B).

#### Grooming episodes

Figure 3C and D present the grooming episode time (Fig. 3C) and the number of episodes of paw licking or short grooming (Fig. 3D) in the OFT arena of animals in the terminal stage of *T. gondii* infection, as well as the impact of ovariectomy, LPD and HFD on these parameters in animals in the early stage of infection. The infected control group and animals fed with LPD displayed significant decreases in grooming time compared with the non-infected group (*P* = 0.006 and 0.008, respectively), but also compared with the infected ovariectomized (*P* = 0.023 and 0.025, respectively) and HFD-fed rats (*P* = 0.019 and 0.021, respectively; Fig. 3C). Moreover, while a marked decrease in the number of episodes of paw licking was observed in terminal stage infected animals compared with the non-infected group (*P* = 0.006), but also compared with early-stage infected control group (*P* = 0.037) and HFD-fed (*P* = 0.002) and ovariectomized (*P* = 0.011) infected groups (Fig. 3D). Of all groups sacrificed at dpi 17, a decrease was only observed in LPD-fed animals, but with high inter-individual differences preventing statistical significance (*P* = 0.725; Fig. 3D).

#### Entries into arena areas

Figure 3E and F present the latency to the first entry (Fig. 3E) and the time spent (Fig. 3F) in the arena centre of animals in the terminal stage of *T. gondii* infection, as well as the impact of ovariectomy, LPD and HFD on these parameters in animals in the early stage of infection. The latency to the first entry to the arena centre was markedly higher in all infected groups than in the non-infected groups, with the lowest increase in infected control animals (*P* = 0.021), a higher increase in the late-stage infected animals (*P* < 0.001) and a high inter-individual variability in ovariectomized animals (*P* = 0.055; Fig. 3E). Moreover, all infected groups displayed marked decreases compared with non-infected groups in the time spent in the arena centre, with *P* < 0.001 for late-stage infected and for LPD-fed groups, *P* = 0.001 for the ovariectomized and *P* = 0.002 for infected control and HFD-fed groups (Fig. 3F). Overall, the most significant decreases in indicators of arena centre activities were observed in the late-stage infected group, and in animals fed with LPD, the less marked decreases were observed in infected control animals, while high inter-individual variabilities were commonly observed in ovariectomized and HFD-fed groups (Fig. 3E and F).

#### SAP and rearing episodes

Figure 3G–I present the SAP time (Fig. 3G) and the number of episodes of rearing against the wall (Fig. 3H) and on hindlimbs (Fig. 3I) in the OFT arena of animals in the terminal stage of *T. gondii* infection and the impact of ovariectomy, LPD and HFD on these parameters in animals in the early stage of infection. Late-stage infected rats showed a significant increase in the SAP total time (*P* < 0.001; Fig. 3G) compared with non-infected rats. Significant increases in SAP time were observed in the early-stage infected control (*P* = 0.003) and in the infected group fed with LPD (*P* = 0.001), HFD (*P* = 0.004) or ovariectomized (*P* < 0.001) compared with the non-infected group (Fig. 3G). Despite this increase, the SAP times of groups sacrificed at early stage were still significantly lower than those of terminal-stage infected animals (*P* < 0.001; Fig. 3G). Instead, compared with non-infected animals, significant decreases in the number of episodes of rearing against the wall were observed in the late-stage infected (*P* < 0.001), in the infected control (*P* = 0.002), in infected fed with LPD (*P* < 0.001) and in ovariectomized (*P* = 0.002) groups, but not in the HFD-fed group (*P* = 0.054; Fig. 3H). Animals fed with LPD had the lowest number of episodes among the groups sacrificed at dpi 17, but the numbers of episodes of rearing against the wall of all groups sacrificed at dpi 17 were still significantly higher than values of late-stage infected animals (*P* = 0.018 versus LPD-fed and *P* < 0.001 versus other infected groups; Fig. 3H). In addition, all infected animals showed markedly lower numbers of episodes of rearing on hindlimbs compared with non-infected animals, with the lowest numbers observed in the late-stage infected animals (*P* < 0.001) and in infected animals fed with LPD (*P* < 0.001; Fig. 3I).

### EPM cognitive and motor indicators

**Figure 4:**
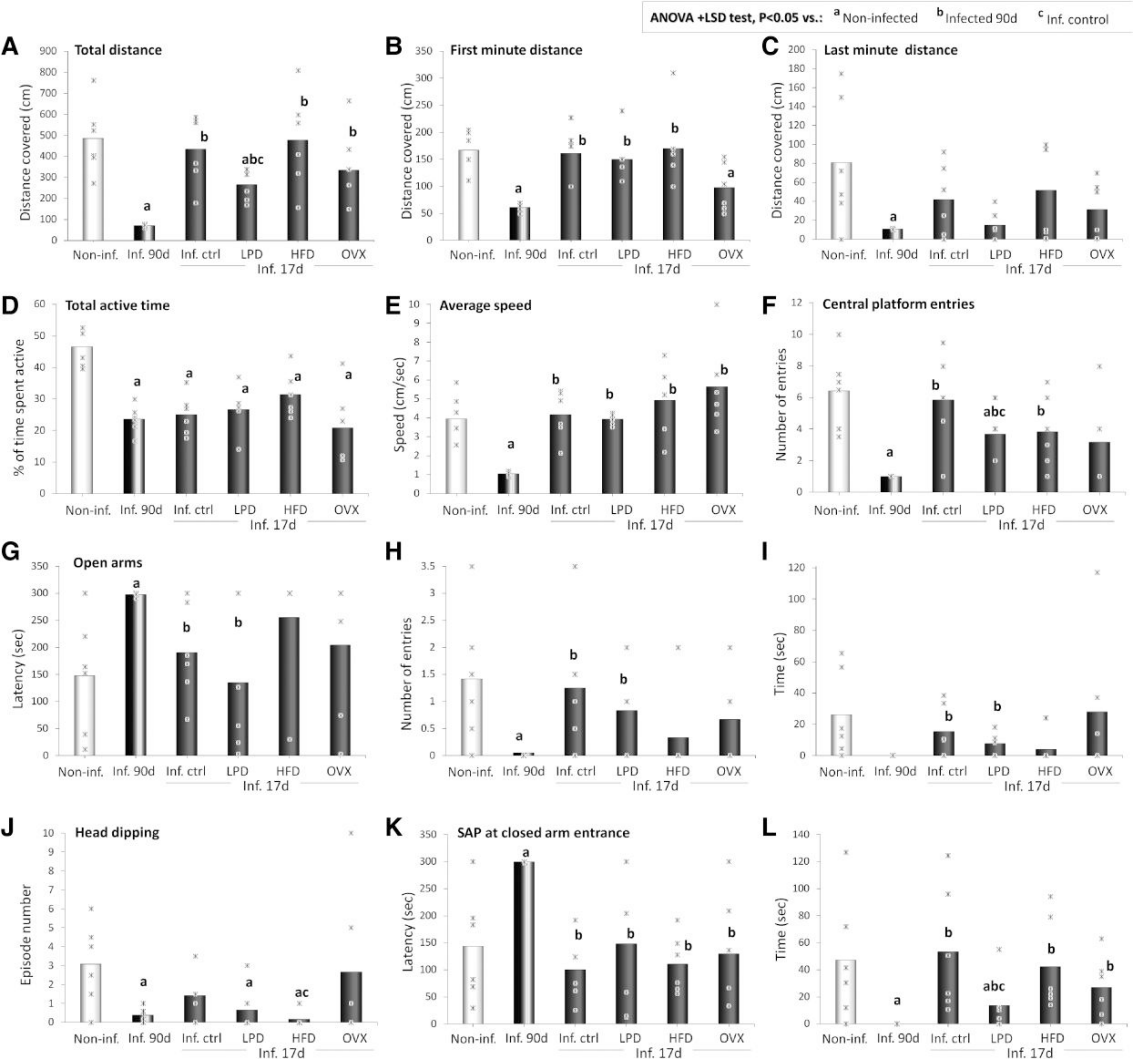
**EPM motor and cognitive indicators.** Distance covered (**A–C**), activity time (**D**) and speed in the maze (**E**), central platform entries (**F**); the latency to first entry (**G**), number of entries (**H**) and time spent (**I**) in the open arms; and numbers of episodes of heading dipping (**J**) and latency (**K**) and time (**L**) of SAP at closed arm entrance of non-infected (non-inf.) animals, *T. gondii*-infected animals at the terminal stage of infection (inf. 90d) and infected animals at the early stage (inf. 17d) fed chow (inf. ctrl), LPD, HFD or ovariectomized (OVX). *n* = 6 animals/group. Critical value of ANOVA *F*-score: 4.35.

Figure 4 presents the total distance covered (Fig. 4A–C), the activity time (Fig. 4D) and speed in the maze (Fig. 4E), central platform entries (Fig. 4F), the latency to first entry (Fig. 4G), the number of entries (Fig. 4H) and time spent (Fig. 4I) in the open arms, as well numbers of episodes of heading dipping (Fig. 4J) and latency (Fig. 4K) and time (Fig. 4L) of SAP at closed arm entrance in the EPM of animals in the terminal stage of *T. gondii* infection, as well as the impact of ovariectomy, LPD and HFD on these parameters in animals in the early stage of infection.

#### Distance covered and speed

Late-stage infected animals, and to a lesser extent the group fed with LPD, displayed significant decreases in the distance covered in the EPM compared with the non-infected (*P* < 0.001 and *P* = 0.010, respectively), with the early-stage infected control (*P* < 0.001 and *P* = 0.004, respectively), and with the HFD-fed groups (*P* = 0.007 and *P* = 0.052, respectively; Fig. 4A). The same trend was observed in the distance covered in the EPM during the first minute of the test (Fig. 4B). Instead, while all infected animals showed decreases in the distance covered in the EPM during the last minute of the test compared with the non-infected, the most marked and only statistically significant decrease was observed in the late-stage infected group (*P* = 0.021; Fig. 4C). Similarly, decreases in the time spent active were observed in the late-stage infected group (*P* = 0.032 versus non-infected group) and comparably in all infected groups sacrificed at dpi 17 (15–25%↓, *P* < 0.05; Fig. 4D). Late-stage infected animals displayed markedly decreased speeds in the maze compared with non-infected animals (*P* < 0.001), but no statistically significant change was observed compared with non-infected in animals sacrifice at dpi 17 (Fig. 4E).

#### Central platform and open-arm entries

The late-stage infected rats, compared with the non-infected, had a significantly lower number of entries into the central platform of the maze (*P* = 0.009; Fig. 4F). The number of entries into the central platform was also decreased in infected animals fed with LPD compared with the non-infected group (*P* = 0.012) and with the infected control group (*P* = 0.006; Fig. 4F). Instead, all infected animals showed increases in open-arm latency compared with non-infected animals, but the most marked and only statistically significant difference was observed in the late-stage infected group (*P* = 0.003; Fig. 4G). Notably, infected groups sacrificed at dpi 17 displayed high inter-individual variabilities and had significantly lower open-arm latencies than the terminal-stage group (Fig. 4G). In addition, all infected animals showed decreases in open-arm entries, with the most marked and only statistically significant difference in the late-stage infected group (*P* = 0.039; Fig. 4H). The LPD-fed and HFD-fed groups also displayed decreases in open-arm entries compared with non-infected and infected control groups, although with high inter-individual variabilities (Fig. 4H). Except for the early-stage infected control and the ovariectomized infected groups, all infected groups showed decreases in open-arm time, although these changes were not statistically significant compared with the non-infected group (Fig. 4I). Significant increases in both open-arm number of entries and time were observed in the early-stage infected control (*P* = 0.016 and 0.037, respectively) and the ovariectomized (*P* = 0.012 and 0.014, respectively) infected groups compared with the terminal stage group (Fig. 4H and I).

#### Head dipping and SAP episodes

Except for the ovariectomized group, all infected groups displayed marked decreases in head dipping episode number compared with non-infected animals, which were statistically significant in the late-stage infected (*P* = 0.010 and 0.021, respectively), in LPD-fed (*P* = 0.014 and 0.039, respectively) and in HFD-fed (*P* = 0.006 and 0.018, respectively) groups (Fig. 4J). Compared with the non-infected group, late-stage infected rats showed a markedly increased latency to the first episode of SAP at closed arm entrance (*P* = 0.001; Fig. 4K). Notably, the SAP latencies of late-stage infected rats were also significantly higher than those of the early-stage infected control (*P* < 0.001), LPD-fed (*P* = 0.002), HFD-fed (*P* < 0.001) and ovariectomized (*P* = 0.012) infected groups (Fig. 4K and L). In addition, significant decreases in SAP time were observed compared with non-infected animals in the late-stage infected group (*P* = 0.008) and in the LPD-fed group (*P* = 0.042; Fig. 4L). The SAP time of the latter was markedly less than the SAP time of all groups sacrificed at dpi 17 (Fig. 4L).

### Stained section observation and neuronal counts

**Figure 5:**
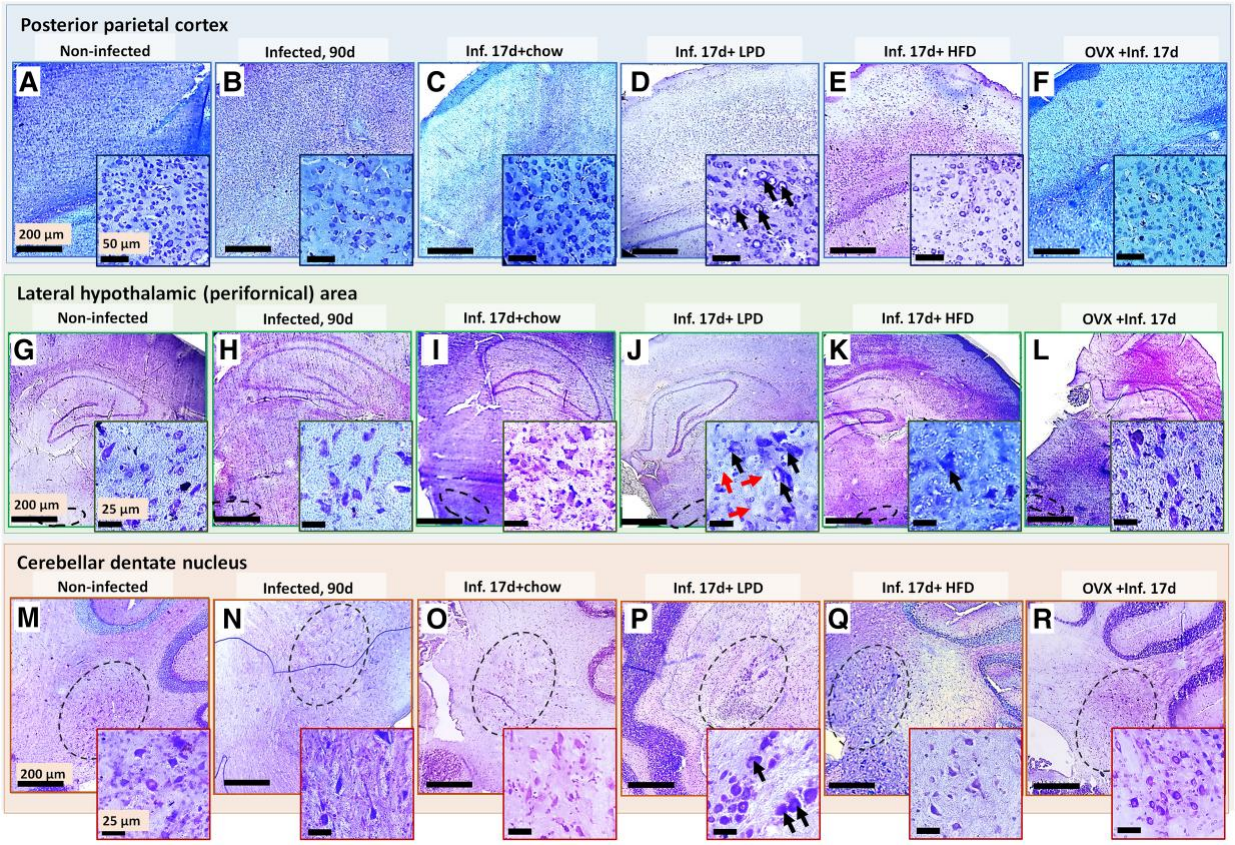
**Nissl-stained brain sections.** Micrographs of cresyl violet–stained brain sections of representative animals of non-infected animals (**A**, **G**, **M**), *T. gondii*-infected groups at the terminal stage of infection (**B**, **H**, **N**) and infected animals at the early stage fed with LPD (**D**, **J**, **P**), HFD (**E**, **K**, **Q**) or ovariectomized (**F**, **L**, **R**). Note the marked decrease in neuronal density in 90-day infected animal brains without indications of missing neuron nuclei as well as the marked decrease in neuronal density accompanied with enlarged neurons and evidence of recent neuron death in 17-day infected, particularly in the LPD-fed animals. Black arrows: enlarged neurons, red arrows: missing neuron nuclei.

**Figure 6:**
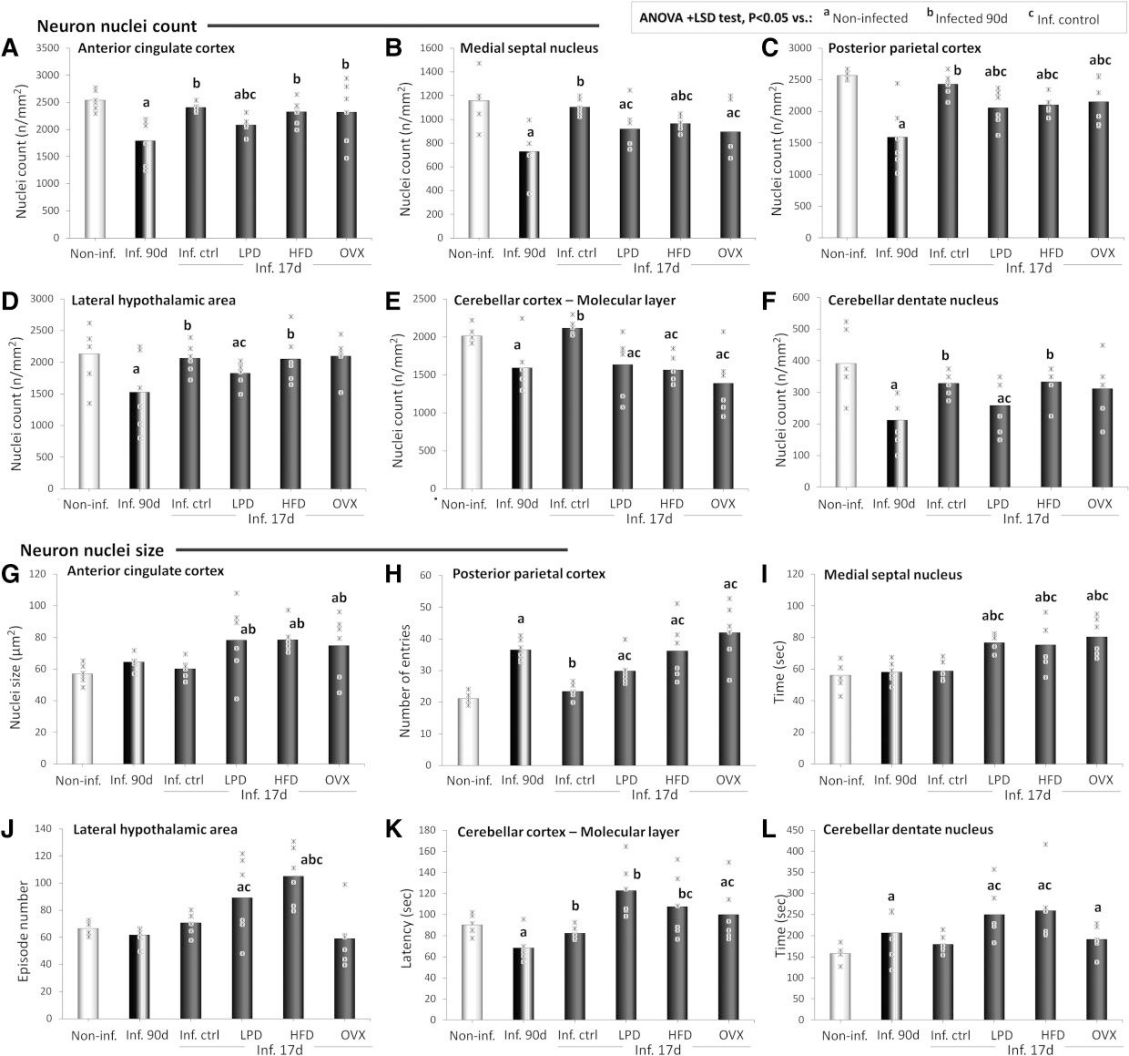
**Brain neuron counts.** Neuron nuclei density (**A–F**) and size (**G**-**L**) in the anterior cingulate cortex (**A** and **G**), medial septal nucleus (**B** and **H**), posterior parietal cortex (**C** and **I**), peri-fornical area (**D** and **J**), cerebellar molecular layer (**E** and **K**) and dentate nucleus (**F** and **L**) of non-infected (non-inf.) animals, *T. gondii*-infected animals at the terminal stage of infection (inf. 90d) and infected animals at the early stage (inf. 17d) either fed chow (inf. ctrl), LPD, HFD or ovariectomized (OVX). *n* = 6 animals/group. Critical value of ANOVA *F*-score: 4.35. *T. gondii*-infected animals at the terminal stage of infection and infected animals at the early stage fed with LPD, HFD or OVX. *n* = 6 animals/group. Critical value of ANOVA *F*-score: 4.35.

Figure 5 shows micrographs of cresyl violet–stained brain sections of representative animals, and Fig. 6 shows the results of brain neuron counting of *T. gondii*-infected animals at the terminal stage of infection and infected animals at the early stage fed with LPD, HFD or ovariectomized. In the posterior parietal cortex, compared with the non-infected rats, terminal stage animals infected showed the most marked decrease in neuronal density among all the groups (Fig. 5B, H and N). LPD-fed infected rats also showed a marked decrease in neuronal density, but in addition, animals of this group presented with enlarged neuron nuclei (Fig. 5D, J and P). To a lesser extent, enlarged nuclei were also observed in the other groups sacrificed in the early stage of the infection (Fig. 5E, F, K, L, Q and R). Instead, in the lateral hypothalamic area, LPD-fed infected rats displayed very enlarged neuron nuclei and a marked decrease in neuron nuclei density compared with the non-infected rats (Fig. 5G and J). To a lesser extent, enlarged nuclei were also observed in the early-stage infected control rats and in HFD-fed and ovariectomized infected rats (Fig. 5K and L). Instead, terminal-stage infected showed a marked decrease in neuron nuclei density (Fig. 5H). Furthermore, compared with the non-infected rats, terminal-stage infected rats presented with a marked decrease in the density of large neuron nuclei, while marked losses in neuron nuclei density were observed in LPD-fed infected rats (Fig. 5M–R). Misshaped nuclei were observed in the early-stage infected controls and in HFD-fed and ovariectomized infected rats (Fig. 5).

Compared with the non-infected rats, the terminal-stage infected rats had significantly lower neuronal counts in the anterior cingulate cortex (*P* < 0.001; Fig. 6A), medial septal nucleus (*P* = 0.001; Fig. 6B), posterior parietal cortex (*P* < 0.001; Fig. 6C), lateral hypothalamic area (*P* = 0.027; Fig. 6D), cerebellar molecular layer (*P* = 0.006; Fig. 6E) and dentate nucleus (*P* < 0.001; Fig. 6F). No marked change in neuronal counts was observed in these brain areas in early-stage infected control animals compared with the non-infected rats (Fig. 6A–F). Instead, decreases in neuronal counts were observed in all the infected groups sacrificed in the early-stage compared with the non-infected rats and with the infected control group in the anterior cingulate cortex (Fig. 6A), medial septal nucleus (Fig. 6B), posterior parietal cortex (Fig. 6C) and cerebellar molecular layer (Fig. 6D). Only LPD-fed rats displayed significant decreases in neuronal count in the dentate nucleus (*P* = 0.046 and 0.039, respectively; Fig. 6E) and, to a lesser extent, in the lateral hypothalamic area (*P* = 0.028 and 0.097, respectively; Fig. 6F).

Except for the infected control rats whose values were comparable with the non-infected rats, all the infected animals sacrificed in the early stage had significantly higher neuron nucleus sizes in the anterior cingulate cortex (Fig. 6G) and in the posterior parietal cortex (Fig. 6H). No significant change in neuronal counts was observed in the terminal-stage infected and in the early-stage infected control groups in these brain areas (Fig. 6G and H). All infected groups had significantly higher neuron nucleus sizes in the medial septal nucleus compared with the non-infected group, except for the infected control group whose values were comparable (Fig. 6I). In addition, the HFD-fed and LPD-fed infected groups had significantly higher neuron nucleus sizes compared with the non-infected, with early-stage infected control and with a lesser extent terminal-stage groups in the lateral hypothalamic area (*P* < 0.001, *P* < 0.001 and *P* = 0.001, respectively, for HFD-fed group and *P* = 0.020, *P* = 0.026 and *P* = 0.063, respectively, for LPD-fed group; Fig. 6J) and in the cerebellar molecular layer (*P* = 0.091, *P* = 0.022 and *P* = 0.004, respectively, for HFD-fed group and *P* = 0.037, *P* = 0.077 and *P* = 0.004, respectively, for LPD-fed group; Fig. 6K). Terminal-stage infected animals displayed significant decreases in neuron nucleus size in the cerebellar molecular layer (Fig. 6K) and increases in the dentate nucleus (Fig. 6L) compared with the non-infected group (*P* = 0.015 and 0.007, respectively). Furthermore, the HFD-fed and LPD-fed infected groups had significantly higher neuron nucleus sizes compared with the non-infected (*P* < 0.001) and with early-stage infected control groups in the dentate nucleus (*P* = 0.002 and 0.004, respectively; Fig. 6L).

## Discussion

The results of this study show marked alterations in the body weight, body temperature, blood glucose level and in indicators of cognitive and motor functions in terminal-stage *T. gondii*-infected rats compared with non-infected rats. Comparable alterations were observed in early-stage LPD-fed rats as discussed later below. *T. gondii*-infected rats displayed increases in body weight until dpi 67, although their growth was slower than that of their non-infected counterparts. Then, increasingly marked losses in body weight and other severe signs of disease were observed, culminating at ∼dpi 90, when the animals were sacrificed. Such alterations were expected, considering that cachexia and other components of infection-associated wasting syndrome are normal signs of terminal-stage toxoplasmosis.^71,72^ The cause of the late occurrence of weight loss and other signs of the disease in immunocompetent laboratory animals, including neuropsychological signs, are controversial, but these signs are commonly attributed to systemic inflammation and reactive neuroinflammation triggered by the burden of latent tissue cysts.^14,15^ Interestingly, compared with animals in the terminal stage of infection, increasingly marked losses in body weight and severe signs of disease were also observed in early-stage infected animals fed with LPD, but not in HFD-fed or in ovariectomized infected animals. In addition, both late-stage infected and early-stage LPD-fed rats had significantly lower blood glucose levels than non-infected control animals or early-stage infected control animals, further supporting the occurrence of wasting syndrome, as altered glucose metabolism is a key component of wasting syndrome.^73,74^ These observations suggest that undernutrition accelerated the course of the disease associated with *T. gondii* infection in rats still at the early stage of infection, possibly due to undernutrition-associated immune system dysfunctions.^22,23^ Notably, in an early study addressing the effect of nutritional deprivation on immune function in mice, starved animals lost their protection against *T. gondii* infection; and even macrophages from these mice lost the ability to prevent *Toxoplasma* trophozoite multiplication *in vitro*.^23^ The ability of LPD to induce terminal-stage–like disease in early-stage infected animals indicates that protein undernutrition may trigger severe neurotoxoplasmosis even before the occurrence of well-established pathological drivers of latent infection–associated neurotoxoplasmosis, such as the persistence of the parasite and deleterious interactions with host immune responses and the resulting systemic inflammation and reactive neuroinflammation.^6,38,39^ In addition, considering that undernutrition is a global problem and is more common in populations more likely to develop active toxoplasmosis, namely HIV patients, pregnant women and infants,^75,76^ it appears that undernutrition has probably favoured the global spread and success of *T. gondii* to the current point where up to a third of the world's population harbours a successful latent infection.^1,2^

Additionally, compared with non-infected and with infected control animals, LPD-fed infected rats displayed strong terminal-stage–like alterations in cognitive and motor functions indicators. Cognitive function impairment indicators observed in the late-stage infected and in early-stage LPD-fed infected animals were mainly: (i) higher latency times to the first entry to the OFT arena centre and to the first exploration of EPM open arms compared with non-infected rats, suggesting a weakening of rat's natural drive to explore novel environments^77,78^; (ii) significant increases in the time spent in SAP in the arena angles, an avoidance response that may suggest an increase in rat anxiety^79,80^; (iii) marked decreases in the activities around the EPM central platform, a component of risk assessment and aversive-state escape responses, which indicate the natural hesitation of animals when choosing the arm to explore in their attempt to avoid stressors^81,82^; (iv) significantly less episodes of head dipping in the EPM, a major indicator of rodent risk assessment abilities^83,84^; and (v) drastic reduction in rearing episode number, which in a novel environment such as OFT arena suggests increased neophobia and could be a prodromal indicator of cognitive disorders.^85^ Indicators of motor function alterations observed in the late-stage infected and in early-stage infected animals fed with LPD included decreases in distances covered in the OFT arena and in the EPM and an altered gait characterized by the tendency to use anterior footpads more and heels less, with significant decreases in stride and step lengths that may suggest neurologic deficits.^86,87^ Notably, stride and step length reductions are commonly observed in neurodegenerative diseases, such as Parkinson's disease, Huntington's disease and their murine models.^87,88^ Altogether these findings may suggest cognitive impairment, in particular, poor risk assessment and aversive-state escape responses in *T. gondii-*infected rats at the terminal stage of infection and indicate that an LPD accelerated neurotoxoplasmosis. Previous reports of comparable findings in terminal-stage *T. gondii-*infected rodents proposed that the parasite would induce this behaviour, for instance through neuroinflammatory processes leading to the modulation of host glutamate and dopamine neurotransmission, to facilitate host predation and its dissemination to other hosts.^15,39,40^

Furthermore, in our study, compared with the non-infected, the infected rats displayed significant decreases in both paw licking (rostral/cephalic grooming) and grooming (complex grooming sequences) episode numbers in the OFT arena, although with a high inter-individual variability. This is surprising, considering that it is widely accepted that rats respond to mild stressors by concomitant decreases in episodes of complex grooming sequences and increases in cephalic grooming episodes.^89,90^ To our knowledge, this is the first report of such atypical alterations. Future studies addressing the neurobiological basis of this alteration may improve our understanding of the pathophysiology of neurotoxoplasmosis, particularly neurological and psychiatric alterations.^91,92^ In addition, the observation of Nissl-stained brain sections in the anterior, posterior and cerebellar areas, critical for cognitive and motor responses, revealed the enlargement of neuron nuclei in various areas in LPD-fed infected animals, but also in the other early-stage infected groups, although to a lesser extent. Marked neuronal losses, as indicated by decreases in neuron nuclei density, were mostly observed in LPD-fed infected animals and in terminal-stage infected animals. Counts of neurons confirmed these observations, with significant neuronal losses in the terminal-stage infected animals and, to a lesser extent, in LPD-fed infected animals in all the brain areas considered. Thus, the acceleration of the infection observed in LPD-fed infected animals emerged at least partly from early neuronal loss in brain areas critical for cognitive and motor responses as for terminal-stage infected animals. The areas assessed included the following: (i) the anterior cingulate cortex and the medial septal nucleus for brain anterior zone sections, (ii) the posterior parietal cortex and around the fornix (peri-fornical area) in the lateral hypothalamic area for the brain posterior zone and (iii) the cerebellar molecular layer and dentate nucleus for the cerebellar zone.^61-64^. For most of the areas assessed, marked neuron nuclei enlargement was observed in LPD-fed infected animals and, to a lesser extent, in ovariectomized and HFD-fed infected animals. These observations suggest that *T. gondii* infection worsened the reportedly mild neuronal loss observed in these rat models of menopause and obesity metabolic syndrome, where brain functional alterations are mostly due to cognitive and plasticity losses and related declines in neuronal activities resulting from hormone imbalance, neuroinflammation and mild oxidative stress.^93-96^. Considering that neurons would increase their nucleus size in an attempt to compensate for loss of function in neurodegenerative diseases and that such changes increase their risk of early death,^97-99^ it can be suggested that that neurons of *T. gondii*-infected animals, in particular LPD-fed ones, possibly died after increasing their size to compensate for the loss of function associated with the infection, which was hinted in this study by significant alterations in behavioural test cognitive function indicators. This occurred early in the infection, so cyst persistence cannot be blamed for these terminal-stage–like brain functional alterations. Indeed, reactive neuroinflammation due to persistent *T. gondii* brain cysts and the resulting neurotransmitter imbalance are widely accepted as the main drivers of late-stage cognitive and motor impairments both in humans^6,38,39^ and in experimental models.^13-15,42^ Therefore, it appears that other mechanisms, which probably also occur concomitantly with cyst persistence in the terminal stage of the infection, may account for these alterations. Considering that the systemic disease is most severe in the week following *T. gondii* infection^14,15^ and that the negative inflection in body weight occurred in our LPD-fed rats at the end of that week, immune-to-brain signalling could be one of these mechanisms.^100,101^

## Conclusion

*T. gondii* infection had a negative impact on the overall well-being of the Wistar rats, as indicated by slower increases in body weight in the early and intermediate stages of the infection and cachexia in the terminal stage accompanied by an increase in body temperature and a decrease in blood glucose level. The performance of late-stage infected rats in ethological tests suggested severe motor impairment, including poor gait quality and shorter stride length. In addition, signs potentially indicative of cognitive impairment were observed, such as weakening of the rat's natural drive to explore novel environments, increases in avoidance responses, poor risk assessment and aversive-state escape responses, which may facilitate host predation and dissemination of the parasite to other hosts. Interestingly, comparable alterations were also observed in early-stage infected rats fed with LPD, suggesting that undernutrition accelerated the course of the disease and that wasting syndrome occurrence alone may lead to neurotoxoplasmosis disease, even before the occurrence of well-established pathological drivers associated with latent infection such as reactive neuroinflammation triggered by the persistence of cysts in the brain. Lower neuron counts and the enlargement of their nuclei were observed in LPD-fed infected rats, suggesting a potential role for these deleterious changes in the LPD-induced acceleration of *T. gondii* infection course. Epidemiological and clinical studies addressing a link between undernutrition, neuronal loss and neurotoxoplasmosis may improve our understanding of the biology of neurotoxoplasmosis.

## Data Availability

Data will be made available upon reasonable request.
